# Bilateral rectal sheath hematomas after low-molecular weight heparin treatment in uremia

**DOI:** 10.1080/0886022X.2017.1305406

**Published:** 2017-03-22

**Authors:** Lu Xu, Lei Liu, Xinjian Li

**Affiliations:** a Department of Hemopurification, Affiliated Hospital of Jining Medical College, Jining, Shandong, China;; b Department of Nephrology, Affiliated Hospital of Jining Medical College, Jining, Shandong, China

**Keywords:** Rectus sheath hematomas, hemodialysis, uremic, low-molecular weight heparin

## Abstract

Rectus sheath hematomas (RSHs) are uncommon. They are usually unilateral and rarely bilateral. In this paper, we report the first case of spontaneous bilateral RSHs in a uremic patient after the administration of the first dose of low-molecular weight heparin during hemodialysis. The most interesting aspect of this case is that the main symptom of RSH in our patient was urinary bladder irritation. We highlight the importance of the prompt diagnosis and management of this medical emergency.

## Introduction

Antiplatelet agents have been extensively used during hemodialyisis for uremia. The most commonly used drug is low-molecular-weight heparin (LMWH), which can prevent coagulopathies in the extracorporeal circulation and ensure that hemodialysis proceeds smoothly. However, the benefits of LMWH do not always outweigh the risks of anticoagulation therapy, as a number of adverse events have been reported. Rectus sheath hematoma (RSH) represents an uncommon complication of anticoagulant therapy that can be misdiagnosed as other causes of abdominal pain.

Here, we report an interesting case of an elderly patient with urinary bladder irritation caused by RSH who received conservative treatment in a timely manner, avoiding further complications. To the best of our knowledge, there have been no previous reports of spontaneous bilateral RSH after the first use of LMWH during hemodialysis.

## Case report

A 60-year-old man was transferred to our department with complaints of chest distress and wheezing for 3 d. The patient reported no significant comorbidities apart from a 3-year history of hypertension. An examination revealed the following: temperature, 36.8 °C; blood pressure, 162/98 mm Hg; pulse rate, 78 beats/min; and respiratory rate, >20 breaths/min. He was thin and had no eyelid edema. The breath sounds over both lungs were rough, and a few moist rales were heard. The abdomen was soft, without tenderness or rebound pain.

The laboratory findings were as follows: serum creatinine, 1283 μmol/L (25–123 μmol/L); blood urea, 40 mmol/L (2.29–7.2 mmol/L); white blood cells, 12.6 × 10^9^/L (3.5–9.5 × 10^9^/L); red blood cells, 4.12 × 10^12^/L (3.8–5.1 × 10^12^/L); hemoglobin, 107 g/L (115–150 g/L); platelets, 269 × 10^9^/L (125–350 × 10^9^/L); B-type natriuretic peptide, 146 pg/mL (0–100 pg/mL); prothrombin time, 12.00 s (10–14 s); activated partial thromboplastin time, 35.50 s (23–35 s); fibrinogen, 4.01 g/L (2–4 g/L); K^+^, 6.2 mmol/L (3.5–5.3 mmol/L); Na^+^, 133 mmol/L (135–145 mmol/L); calcium, 2.05 mmol/L (2.2–2.7 mmol/L); phosphorus, 2.13 mmol/L (0.5–1.5 mmol/l); and parathyroid hormone, 472 pg/mL (16–65 pg/mL). Tumor marker, thyroid-function, chest X-ray, and electrocardiographic assessments yielded normal results. Chest computed tomography (CT) revealed inflammatory areas in both lungs and bilateral pleural effusion. Urinary tract color ultrasonography showed that the left kidney measured 9.0 cm × 5.1 cm and had 1.2-cm-thick parenchyma; the right kidney measured 8.8 cm × 5.0 cm, and had 1.3-cm-thick parenchyma. No abnormalities were observed in the ureters or bladder. Owing to the risk of cardiac arrest because of hyperkalemia, hemodialysis was performed. The right femoral vein was catheterized under local anesthesia, and 5000 IU low-molecular weight heparin was administered for anticoagulation.

Four hours after LMWH was administered, the patient felt increasing pain in the lower abdominal region after straining at stool and an urgent desire to micturate. A physical examination revealed a tender mass in the lower abdomen. Urgent bedside urinary tract ultrasonography showed large amount of fluid in the bladder. A 20-Fr in-dwelling catheter was inserted, but there was no obvious outflow of urine through the catheter, and the abdominal pain was not improved. Therefore, abdominal and pelvic CT was performed, which revealed marked hematoma that extended to the prevesical space and bilateral rectus sheath hematomas (RSHs; type III). The following emergency measures were undertaken immediately: bed rest, ice bag application, and compression bandaging. In addition, a blood coagulation profile was obtained, which showed the following: prothrombin time, 12.80 s (10–14 s); activated partial thromboplastin time, 47.40 s (23–35 s); and fibrinogen, 3.04 g/L (2–4 g/L). Given the abnormal coagulation function and risk of continuing hemorrhage, we transfused the patient with 180 mL fresh frozen plasma, supplemented with the injection of 2 U hemocoagulase agkistrodon for hemostasis and 2 U red blood cell suspension.

We then performed heparin-free continuous renal replacement therapy (CRRT) 3 times per week, and closely monitored the patient. After 1 week, a repeat CT showed that the hematoma had not expanded. After six sessions of heparin-free CRRT, the hematoma was slightly reduced, and did not recur when 2500 IU LMWH was administered for hemodialysis. We then increased the heparin dose to 5000 IU without further complications. A follow-up clinical examination 6 months later showed that the abdominal mass had completely resolved.

## Discussion

Our patient was initially misdiagnosed with urinary retention. The urgent need to urinate in the patient was caused by bladder-wall stimulation by the bilateral RSH. Ultrasound has a poor ability to discriminate the bleeding site from the surrounding organs, and thus, led to the incorrect detection of a large amount of sediment in the bladder. This shows that the findings of auxiliary examinations should be interpreted with care. Our patient had no history of trauma, but he had a pulmonary infection and a cough. This combined with straining at stool increased the intra-abdominal pressure. The increased pressure and the use of anticoagulation probably led to abdominal bleeding in our patient. Furthermore, in patients with chronic renal failure and uremia, platelet adhesion and aggregation are abnormal, mainly caused by reduced A2 thromboxane production as a result of abnormal platelet arachidonic acid metabolism.^1^ These are the causes of spontaneous RSH in our patient.

RSH is an uncommon and often misdiagnosed disease. About 60% of RSHs occur in the right lower abdomen^2^ mainly because the posterior sheath below the arcuate line consists of only a thin layer of transversalis fascia, which separates the rectus abdominis muscle from the peritoneum.^3^ RSH occurs more often in women aged 50–60 years than in men, with a female:male ratio of 2–3:1.^4^^,^^5^ Keeping in mind that women have less muscle mass and that pregnancy has been reported as a predisposing factor for RSH, the aforementioned ratio can be explained.^6^ RSH is manifested by a sudden abdominal pain and local mass. Other symptoms depend on the bleeding site and hematoma size. In addition, large hematomas can rarely cause urinary tract obstruction and bladder irritability, or even abdominal compartment syndrome.^6^^,^^7^ Physical examination can help discover the abdominal mass, which becomes more apparent and painful because of muscle tension. In some patients, color changes in the abdominal skin are seen due to abdominal wall bleeding. This is called Cullen sign, and explains why the disease is sometimes misdiagnosed as acute pancreatitis. Because the clinical symptoms of RSH are not typical, it is often misdiagnosed as appendicitis, acute pancreatitis, urine retention, intestinal obstruction, ovarian cyst torsion, etc. The clinical symptoms of abdominal wall hematoma are similar to those of intraperitoneal acute abdomen, and, therefore, require detailed history taking and physical examination. The typical abdominal wall mass can display the Fothergill sign. This sign can also be positive in patients with an abdominal mass.^8^

CT is the preferred method for RSH diagnosis. It can clearly show the location of bleeding and the extent of the hematoma, facilitating the classification and treatment of RSH. Based on CT findings, RSH can be classified as follows:^3^ in Type I RSH, the bleeding is intramuscular with minimal hemodynamic compromise; in Type II, there is blood between the muscle and the transversalis fascia; and in the more severe Type III, there is a marked hematoma that extends to the prevesical space and sometimes intraperitoneally. Ultrasonography is helpful for a rapid diagnosis, and has many advantages such as no radioactivity, timeliness, effectiveness, convenience, and practicability. However, it is operator dependent, which can lead to misdiagnosis. Therefore, the sensitivity and specificity of CT are better than those of ultrasonography. In patients with high probability for RSH and no contradiction, CT should be the first-line diagnostic imaging test.^6^

Most RSH patients gradually recover with hematoma absorption after conservative treatments such as rest, cessation of anticoagulation therapy, and transfusion of blood products when necessary. Surgical treatment is only used in patients with shock because of massive bleeding. This is because surgery has limited effectiveness in RSH. It is difficult to accurately identify the bleeding vessels, and uncontrolled bleeding increases the associated mortality. The mortality rate of RSH is about 4%, but in iatrogenic cases, it is 18%, and in cases of RSH induced by anticoagulants, it can even reach 25%.^9^ Studies have indicated that RSH in the lower abdomen is usually caused by the rupture of the inferior epigastric artery. In an emergency, ligation of this artery may help to prevent shock caused by massive bleeding as well as secure more time for further surgical treatment.^3^
